# Myeloid landscape of BRAF-mutant papillary thyroid cancer and thyroiditis

**DOI:** 10.1530/ERC-26-0088

**Published:** 2026-08-31

**Authors:** Sumathy Perampalam, Matti Gild, Adwoa Amoakowa Sey, Lyndal J Tacon, Mitali Fadia, Cathal King, Alexander Papachristos, Mark Sywak, Anthony J Gill, Martyn Bullock, Roderick J Clifton-Bligh

**Affiliations:** ^1^ Cancer Genetics Laboratory, Kolling Institute of Medical Research, Sydney Medical School, Faculty of Medicine and Health, University of Sydney, Camperdown, New South Wales, Australia; ^2^ The Canberra Hospital, Department of Endocrinology, Canberra, Australian Capital Territory, Australia; ^3^ Australian National University, School of Medicine and Psychology, Garran, Australian Capital Territory, Australia; ^4^ Royal North Shore Hospital, Department of Endocrinology and Diabetes, St Leonards, Australia; ^5^ The Canberra Hospital, Department of Pathology, Garran, Australian Capital Territory, Australia; ^6^ South Australian Immunogenomics Cancer Institute (SAiGENCI), Adelaide University, Adelaide, South Australia, Australia; ^7^ University of Sydney Endocrine Surgery, Faculty of Medicine and Health, University of Sydney, Camperdown, Australia; ^8^ Royal North Shore Hospital, Department of Endocrine Surgery, St Leonards, Australia; ^9^ NSW Health Pathology, Department of Anatomical Pathology, Royal North Shore Hospital, St Leonards, New South Wales, Australia; ^10^ Sydney Medical School, University of Sydney, Camperdown, New South Wales, Australia

**Keywords:** single-cell sequencing, transcriptomics, BRAF, thyroid cancer, thyroiditis

## Abstract

Papillary thyroid cancer (PTC) is less aggressive when associated with lymphocytic thyroiditis (LT), even in the presence of oncogenic *BRAF*, including smaller tumours, less lymph node involvement and reduced extrathyroidal extension. To investigate possible immune mechanisms underlying this association, we compared the tumour microenvironment of PTC-BRAF with LT and that without LT using single-cell RNA sequencing (scRNA-seq). Single-cell libraries were generated from fresh and fixed tumour samples with post-dissociation viability >70% using the 10x Genomics Chromium Platform and sequenced on an Illumina NovaSeq 6000. We analysed scRNA-seq data from 11 PTC-BRAF tumours: four with LT (one publicly available sample) and seven without LT. Downstream analyses included quality control, batch correction, dimensionality reduction, and differential gene expression analysis. We found that neutrophils were the predominant myeloid cell type in PTCs without LT. Thyrocytes without LT showed significant expression of the neutrophil recruitment chemokine *ECRG4*. In the absence of LT, neutrophils expressed oncogenic genes with poor clinical outcomes. In contrast, thyrocytes from tumours with LT showed increased expression of MHC-II antigen presentation, consistent with effective immune surveillance. Thyrocytes and macrophages in the presence of LT showed enrichment of interferon gamma response pathways. Our data suggest that LT in thyroid cancer is associated with enhanced antigen presentation and fewer features of pro-tumourigenic innate immune activity. These results identify previously under-recognised innate immune cell population and associated transcriptomic features, which suggest new mechanisms to target immune treatments in PTC refractory to other therapies.

## Introduction

Papillary thyroid cancer (PTC) is the most common endocrine malignancy, with increasing incidence over the past two decades (1). Although most PTCs have favourable prognosis, tumours harbouring *BRAF* mutations (PTC-BRAF), approximately 60%, are associated with a more aggressive clinical course than *BRAF* wild-type PTCs (2). Part of this difference may be due to the evasion of PTC-BRAF from immune surveillance; although the role of mutant *BRAF* in modulating immune composition in PTC is not well known, it has been shown to play a major role in modulating response to immunotherapy in anaplastic thyroid cancer (ATC) (3, 4). This study examines how the tumour microenvironment and immune surveillance operate in PTC-BRAF, seeking to clarify the mechanisms that may underlie its behaviour and response to therapy.

Inflammation of the thyroid gland, typically lymphocytic thyroiditis (LT), is found in about 30% of patients of PTC (5). Favourable outcomes seen in thyroid cancers associated with LT suggest that this may be a natural model of effective immune response against cancer (6). However, many questions remain, including the mechanisms by which LT might retard cancer growth (or conversely, whether its absence might promote cancer aggression) and whether adaptive or innate immune responses are primarily involved.

Mechanisms of cancer immune surveillance, also relevant in PTCs, may include i) successful antigen presentation and recognition, ii) tumour-induced immune activation, iii) tumour microenvironment (TME) modulation and iv) immune check point regulation (7). Innate immune response represents the first line of defence against cancer and has a major role in activating adaptive immunity. Cells of the innate immune response, including macrophages, neutrophils and dendritic cells (DC), have a broad scope to recognise patterns in TME, as opposed to the antigen-specific immune responses of adaptive immunity comprising T cells, B cells and natural killer cells (8). Macrophages are known to be key regulators of TME. Antagonistic polarity between pro-inflammatory M1 and anti-inflammatory M2 macrophages, which have a pro-tumoural role, is recognised. The impact of neutrophils in TME on PTC is under-studied, and prognostic significance is unclear. Understanding the essential signalling pathways involved in cancer surveillance from a natural immune-effective model can serve to identify novel therapies, including immune checkpoint inhibitors, cancer vaccines or adoptive T cell approaches.

We have shown in a previous meta-analysis that the presence of LT in PTC-BRAF is associated with a less aggressive disease with smaller tumours, less lymph node involvement and less extrathyroidal extension (9). The aim of this study was to compare changes in immune cell populations between PTC-BRAF with LT and PTC-BRAF without LT using single-cell transcriptomics to obtain critical insights into mechanisms of either immune control or evasion, respectively. We hypothesised that in PTC-BRAF, the presence of LT modifies the TME in a manner that might explain improved outcomes, and conversely, the absence of LT would be associated with features consistent with reduced immune cancer surveillance. In this paper, we have focussed on myeloid cells particularly, since relatively little is known about the role of macrophages or neutrophils in thyroid cancer (10). It is not well established whether these innate immune cells have properties that are either immune responsive or evasive, either of which is fundamental to cancer progression.

## Methods

### Sample collection

This study was approved by the research ethics committee. All patients provided written informed consent after full explanation of the purpose and nature of all procedures used. Fresh PTC tissue samples of 1–2 cm diameter were collected in 10 mL RPMI 1640 medium (Thermo Fisher Scientific, USA) from patients who underwent surgery. *BRAF V600E* status was determined by mutation-specific immunohistochemistry (IHC) using clone VE1 (11). Ten *BRAF*-positive samples were carried forward for single-cell analysis.

### Tissue dissociation and single-cell preparation

Tumours were processed immediately after resection. Specimens were minced into 1- to 2-mm fragments and enzymatically dissociated using the Tumour Dissociation Kit – human (Miltenyi Biotec, Germany) with mechanical processing on a gentleMACS Dissociator (Miltenyi Biotec) according to the manufacturer’s recommendations. Enzymatic digestion was performed at 37°C with gentle mixing for up to 40 min, with visual inspection every 10 min to prevent overdigestion. Digestion was quenched with the addition of 1 mL fetal bovine serum.

Cell suspensions were filtered through a 70-μM cell strainer and centrifuged at 350 *g* for 7 min at 4°C. Red blood cells were depleted using the EasySep RBC Depletion Reagent (Stemcell Technologies, Canada) according to the manufacturer’s protocol. Viable cells were purified by fluorescence-activated cell sorting using acridine orange/propidium iodide staining.

One sample processed using Chromium Flex Gene Expression chemistry was fixed using the Next GEM fixation protocol (10x Genomics, USA) and stored at −80°C until capture. All other samples were captured within 24 h of tissue dissociation.

### Single-cell RNA library preparation, sequencing and data processing

Single-cell libraries were generated from suspensions with post-dissociation viability exceeding 70% using the 10x Genomics Chromium Platform according to the manufacturer’s protocols. Seven samples were processed using Next GEM Single Cell 3′ v3, two samples with GEM-X Single Cell 3′ v4 and two samples with Chromium Flex Gene Expression chemistry. Libraries were sequenced on an Illumina NovaSeq 6000, with all capture, library preparation and sequencing steps performed by the Garvan Institute Genomics Platform. FASTQ files were generated using bcl2fastq (Illumina, USA). For 3′ Gene Expression libraries, alignment, barcode processing and UMI counting were performed using Cell Ranger Count 9.0.1 (10x Genomics) with the GRCh38-2024-A reference including intronic regions. The sample processed using Chromium Flex Gene Expression chemistry was processed using Cell Ranger Multi version 9.0.1 (10x Genomics), as the library was generated as part of a multiplexed experiment, although only this sample was included in the present study.

To expand the cohort and enable integrative analysis, publicly available single-cell RNA sequencing data generated using 10x Genomics 3′ Gene Expression v3 chemistry from one additional tumour sample were retrieved from the Gene Expression Omnibus (GEO) under series accession GSE281736, sample GSM8627209.

### Cell type annotation

Cell type annotation was performed using Cellarium Cell Annotation Service (CAS) integrated within Cell Ranger (v9). Probabilistic cell identity labels were assigned using ontology-aware reference mapping against single-cell reference data sets curated through the CZ CELLxGENE census. Annotations were further refined using phenotype-based expression scores, reclassifying epithelial cells with high thyroid differentiation scores as thyrocytes and similarly refining myeloid labels with a neutrophil gene score.

### Primary data processing and quality control

Primary data processing and quality control were performed in a staged manner to mitigate technical artefacts. Exploratory analyses identified low-level ambient RNA contamination across most cell types, dominated by transcripts of B cell and, to a lesser extent, T cell origin, with the strongest effects observed in lymphocytic thyroiditis-positive samples.

Ambient RNA was identified across samples and corrected using SoupX, applied separately to each sample using matched raw and filtered feature–barcode matrices. Clusters for contamination estimation were generated using SCTransform normalisation, principal component analysis, nearest-neighbour graph construction using the first 30 principal components and graph-based clustering. These clusters were supplied to SoupX to enable automated, sample-specific estimation of contamination fractions, with correction applied conservatively to preserve biological signal.

Following ambient RNA correction, standard quality control metrics were computed for each cell, including total UMI counts, number of detected genes, proportion of mitochondrial transcripts and haemoglobin gene expression. Quality control filtering was performed using a coarse cell-type-aware strategy. Within each coarse cell-type group, median absolute deviation-based thresholds were applied to log-transformed UMI and gene counts, excluding cells more than three median absolute deviations below the group median. Mitochondrial transcript proportions were filtered using an upper threshold of five median absolute deviations above the group median, with an additional global hard cap of 20%. Cells with elevated haemoglobin gene expression were removed to eliminate residual erythrocyte contamination. Putative doublets were identified using Scrublet and removed. Minor cell populations comprising fewer than 100 cells across the full data set were removed from downstream analyses.

### Data integration and batch harmonisation

Batch harmonisation was performed in accordance with 10x Genomics’ recommended integration strategies for multi-chemistry data sets. Expression matrices were log-normalised prior to integration. Highly variable genes were identified across the combined data set, and dimensionality reduction was performed using principal component analysis.

Batch correction was applied using Harmony, with sample identity and library chemistry specified as grouping variables. The resulting harmonised embeddings were used for neighbourhood graph construction, clustering and low-dimensional visualisation.

Batch-corrected representations were used exclusively for visualisation, clustering and estimation of cell-type proportions, while uncorrected expression values were retained for differential expression and pathway analyses to preserve biological signal.

### Differential gene expression and pathway enrichment analysis

For each cell type, raw counts were aggregated per sample to generate pseudobulk profiles. Differential expression was assessed using DESeq2, with cohort as the primary factor. Sex-linked genes were excluded prior to model fitting. Differentially expressed genes were defined by adjusted *P* < 0.05 and absolute fold-change threshold greater than 1.5.

Gene set enrichment analysis was performed by ranking genes according to differential expression statistics against curated pathway collections from MSigDB. Significantly enriched pathways were identified based on normalised enrichment scores and adjusted *P* values.

### Spatial transcriptomics: tissue microarray preparation and construction of cDNA libraries and sequencing

Tissue microarrays of 0.6-mm discs per tissue from thyroid cancer paraffin tissue blocks were mounted onto 10x Genomics Visium slide and processed according to the manufacturer’s instructions. Reverse transcription was performed on the slides to synthesise the first-strand cDNA, which was then quantified and subjected to generate the libraries according to the standard workflow provided in the manufacturer’s protocol. Spatial alignment of tissue H&E image and barcode location was created (10x Genomics). The aligned tissue region barcode and sequence files were then processed and aligned to hg38 human transcriptome (UCSC) using the SpaceRanger v1.2.1 pipeline (10x Genomics).

### Spatial transcriptomics: data analysis

Seurat module and Ucell module scores were used to assess specific cell types with spatial feature plots for visualisation.

### Immunohistochemical assessment

Surgical specimens from PTC-BRAF with and without LT were evaluated with IHC staining using the semi-quantitative scoring system, which classifies protein expression based on the percentage of positive cells and the staining intensity. Five random visual fields were selected from each section. Staining area was scored as 0 for ≤5%, 1 for 5–25%, 2 for 26–50%, 3 for 51–75% and 4 for > 75%. Comment on central and peripheral location was made if an obvious difference in distribution was seen. Staining markers were as follows: CD15 for myeloid-neutrophil, CD68 for macrophage and MPO for pan-myeloid.

## Results

### Thyroid tumour single-cell dataset generation

**Figure 1:**
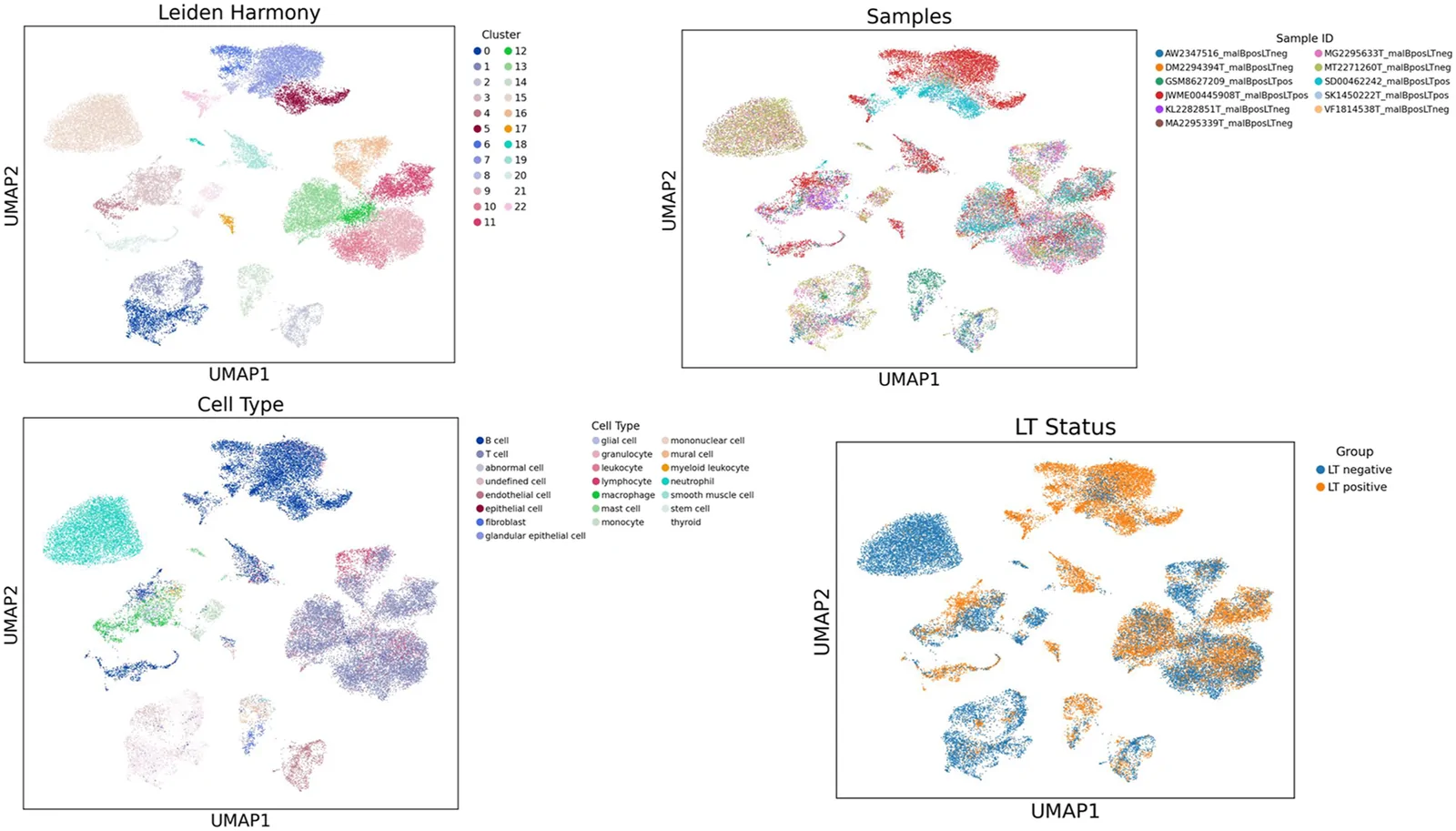
Single-cell atlas: UMAP of (A) cell clusters, (B) samples, (C) cell type and (D) LT status. A full colour version of this figure is available at https://doi.org/10.1530/ERC-26-0088.

We analysed single-cell RNA sequencing (scRNA-Seq) data from 11 PTC-BRAF samples, comprising 10 freshly resected surgical thyroid specimens and publicly available data from 1 PTC-BRAF sample. Four samples were classified as PTC-BRAF with LT (LTpos), and seven samples were classified as PTC-BRAF without LT (LTneg). Annotated histopathological and clinical data are shown in Supplementary Table 1 (see section on Supplementary materials given at the end of the article). Following standardised protocol of quality control filtering and doublet removal, data from all samples were subsequently merged and integrated to account for patient-specific and technical variability. Batch correction was visually assessed and demonstrated robust inter-sample integration with no residual batch effect. After filtering, a total of 19,355 cells in LTpos and 19,312 cells in LTneg samples were identified. Batch-corrected dimensionality reduction and unsupervised clustering identified 22 transcriptionally distinct clusters, representing the major immune, stromal and epithelial compartments of the thyroid TME. Initial coarse cell-type annotation was performed by Cellarium Cell Annotation Service, providing ontology-aware assignments including epithelial cells, B cells, T cells and granulocytes. Further refinement to identify biologically plausible thyroid and granulocytic populations was performed using thyroid and neutrophil differentiation scores (2) (Fig. 1).

### Cell proportions show preponderance for myeloid cells in LT-negative PTCs

**Figure 2:**
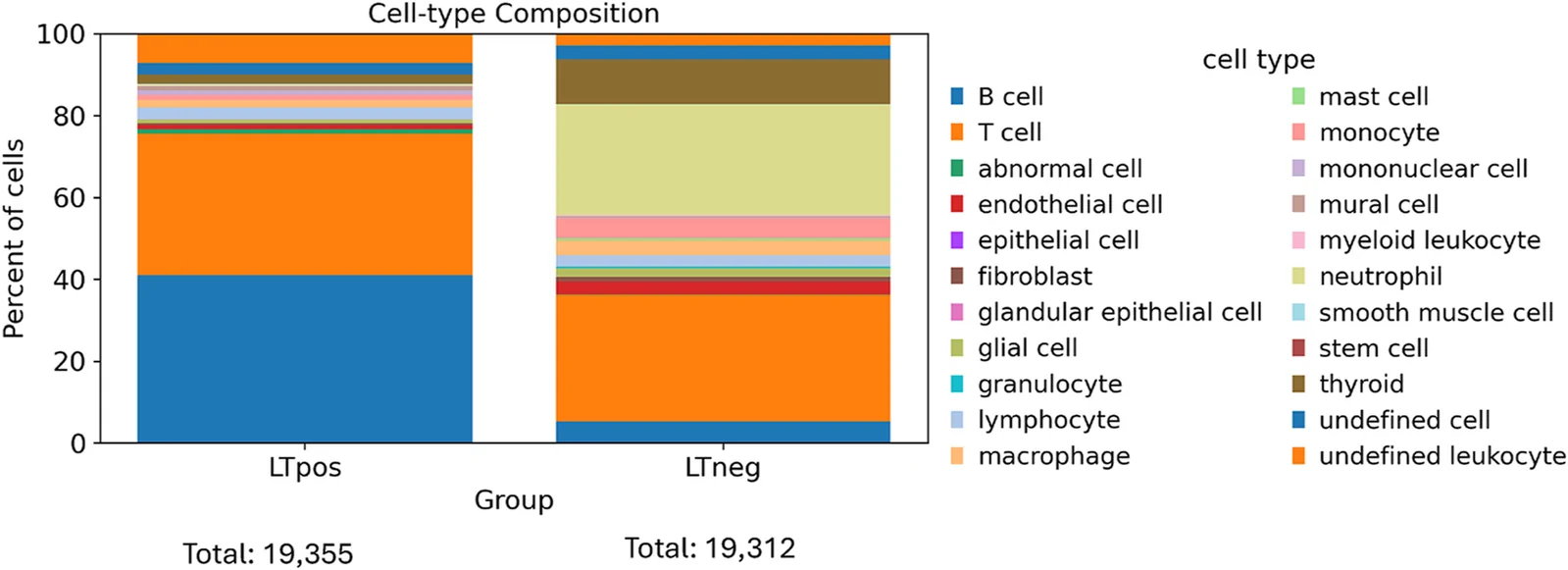
Cell counts: BRAFposLTpos compared with BRAFposLTneg. A full colour version of this figure is available at https://doi.org/10.1530/ERC-26-0088.

LTpos samples demonstrated a significantly reduced portion of thyrocytes, accounting for 2.8% of total cells, compared with 9.1% in LTneg samples, consistent with increased lymphocytic infiltration in lymphocytic thyroiditis (Fig. 2).

Immune cell characterisation revealed significantly more lymphocytes than myeloid cells in LTpos PTCs, whereas LTneg PTCs had significantly more neutrophils. Specifically, in LTpos PTCs, B lymphocytes comprised 32.4% of cells, T cells 43.2%, macrophages 1.9% and neutrophils 0.8%. In contrast, in LTneg PTCs, B cells accounted for only 5.1% of cells but neutrophils were 20.5% of cells; T cells (30.1%) and macrophage (6.1%) proportions were comparable to LTpos PTCs (Table 1).

**Table 1:** Cell counts: BRAFposLTpos compared with BRAFposLTneg.

Cell type	LTpos samples (*n*)	LTneg samples (*n*)	LTpos mean PTC	LTneg mean PTC	Delta mean PTC	MWU stat	*P *value
Monocyte	4	7	0.866	5.334	−4.468	1.0	0.012121
Neutrophil	4	7	0.790	20.522	−19.733	2.0	0.024242
Mast cell	4	7	0.031	0.338	−0.307	4.0	0.070005
B cell	4	7	32.369	5.049	27.320	24.0	0.072727
Myeloid leucocyte	4	7	0.136	0.507	−0.371	4.0	0.072727
Thyroid	4	7	2.805	9.117	−6.312	4.0	0.072727
Glial cell	4	7	0.505	4.577	−4.072	6.0	0.163636
Abnormal cell	4	7	0.847	0.141	0.706	21.5	0.175734
Granulocyte	4	7	0.098	0.430	−0.332	6.5	0.184875
Glandular epithelial cell	4	7	0.002	0.122	−0.120	7.5	0.216269
Undefined cell	4	7	3.374	3.279	0.094	7.0	0.230303
T cell	4	7	43.206	30.145	13.061	20.0	0.315152
Stem cell	4	7	0.006	0.020	−0.014	10.0	0.443624
Endothelial cell	4	7	2.006	4.417	−2.411	10.0	0.527273
Mural cell	4	7	1.263	0.394	0.869	17.0	0.606404
Smooth muscle cell	4	7	0.431	0.185	0.246	17.0	0.606404
Lymphocyte	4	7	3.223	2.568	0.654	17.0	0.648485
Macrophage	4	7	1.934	6.145	−4.211	11.0	0.648485
Epithelial cell	4	7	0.009	0.118	−0.109	15.0	0.912853
Fibroblast	4	7	0.563	1.464	−0.901	14.0	1.000000
Mononuclear cell	4	7	0.718	0.390	0.328	14.0	1.000000
Undefined leucocyte	4	7	4.818	4.737	0.082	13.5	1.000000

### Thyroid cell characterisation reveals enhanced antigen presentation in LTpos PTCs and LTneg PTCs with tumour-promoting effect

**Figure 3:**
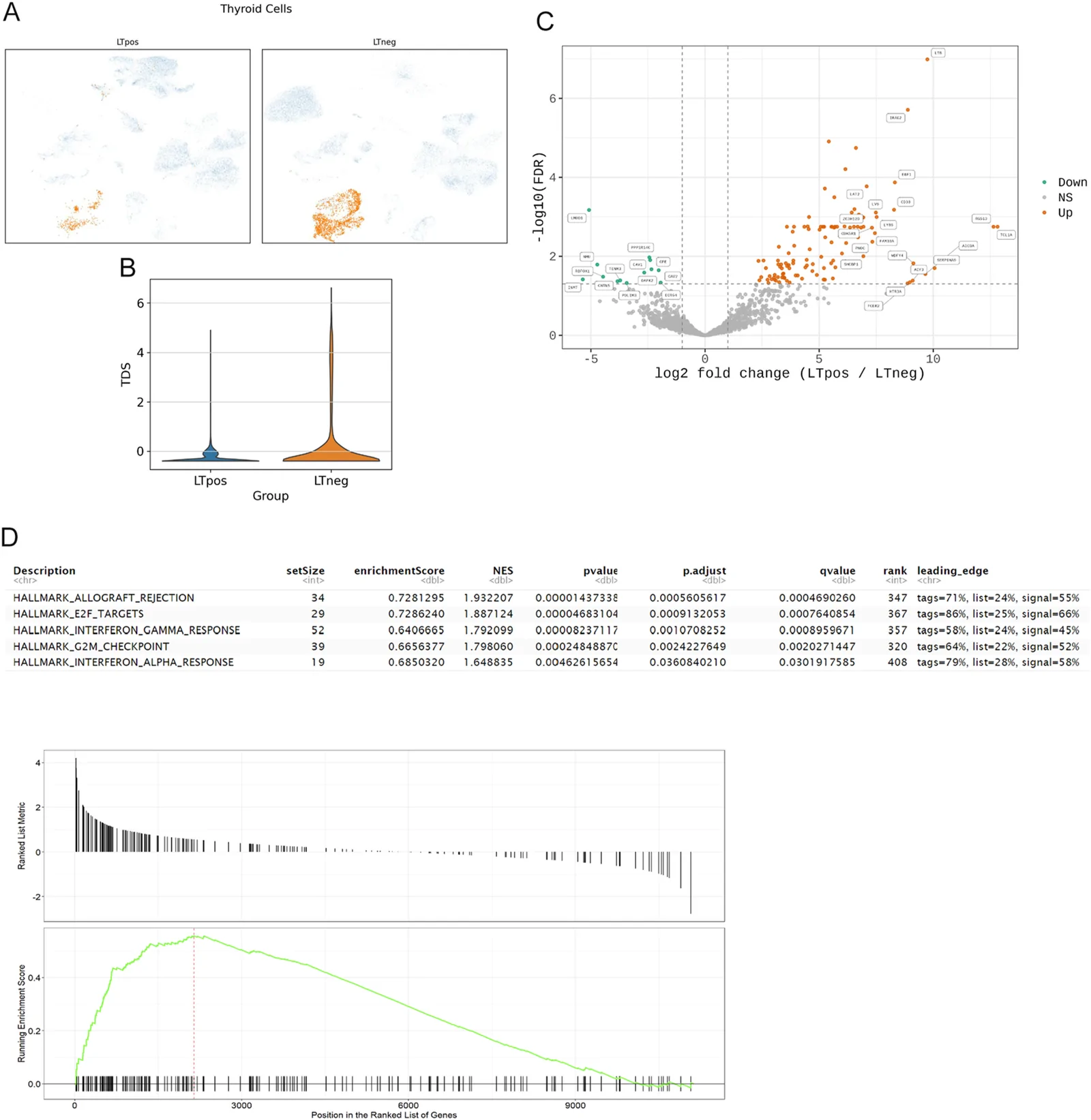
(A) Thyroid cell clusters; (B) thyroid cell violin plots; (C) thyroid cell volcano plots: BRAFposLTpos compared with BRAFposLTneg; (D) thyroid cells: GSEA BRAFposLTpos compared with BRAFposLTneg. A full colour version of this figure is available at https://doi.org/10.1530/ERC-26-0088.

Thyrocytes were identified by thyroid differentiation score (TDS)-based stratification on the integrated UMAP embedding (Fig. 3A). The TDS distributions were comparable between LTpos and LTneg tumours, indicating preserved thyroid differentiation across the groups (Fig. 3B). Comparative pseudobulk differential expression analysis of thyrocytes from LTpos and LTneg tumours revealed marked transcriptional differences. LTpos thyrocytes demonstrated upregulation of genes involved in immune activation and antigen presentation, including *LTB, CD74, CIITA, HLA-DQA1, EBF1, LAT2* (Log2FC 5.4–9.7, padj < 0.05). These transcriptional changes are consistent with enhanced interferon-driven immune crosstalk within the tumour epithelium (Fig. 3C) (https://www.ncbi.nlm.nih.gov/gene/4050#; https://www.proteinatlas.org/ENSG00000196735-HLA-DQA1; https://www.proteinatlas.org/ENSG00000086730-LAT2; 12, 13, 14, 15). In contrast, LTneg thyrocytes increased expression of genes associated with tumour progression and altered epithelial states, including *LMOD1, PPP1R14C, CPE, NMU, CAV1, CAV2, DAPK2, TENM3, CAV1* and *ECRG4* (Log2FC −5.1 to −1.94, padj < 0.05), several of which have previously been implicated in thyroid carcinogenesis and tumour-promoting phenotypes (16, 17, 18, 19, 20, 21, 22, 23, 24). Notably, *ECRG4* has been linked to the regulation of neutrophil recruitment in tissue injury (25).

Gene set enrichment analysis demonstrated significant enrichment of interferon gamma response (normalised enriched ratio (NES): 1.79, padj < 0.05) and interferon alpha response (NES: 1.64, padj < 0.05) pathways in LTpos thyrocytes (Fig. 3D; Supplementary Table 2A and B). These findings support a model in which LTpos tumours retain a more immunologically engaged thyrocyte phenotype, whereas LTneg tumours exhibit transcriptional programmes associated with epithelial remodelling and tumour progression.

### Macrophages express immune-effective genes in LTpos PTCs and immunosuppressive genes in LTneg PTCs

**Figure 4:**
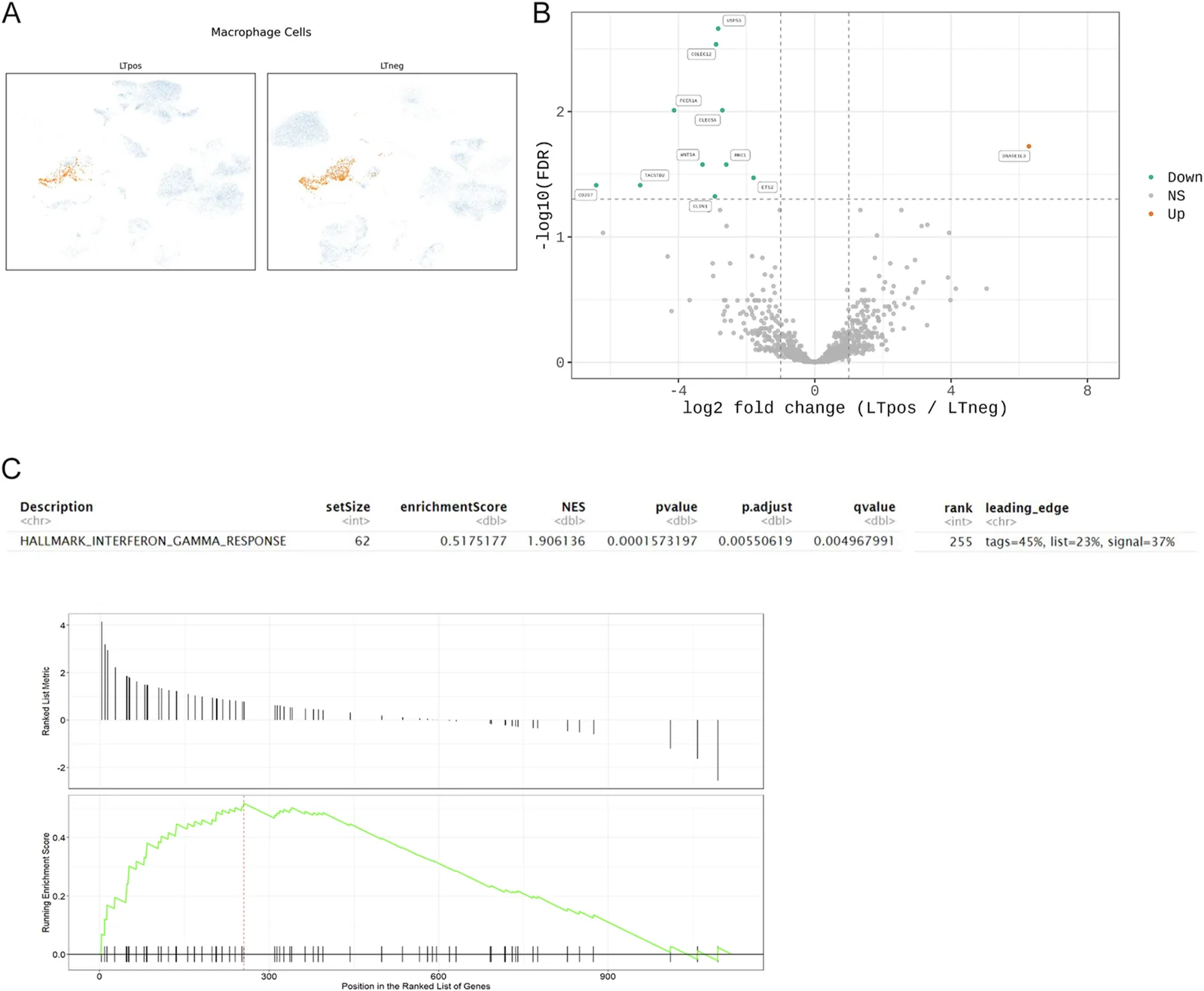
(A) Macrophage cell clusters; (B) macrophage volcano plots: BRAFposLTpos compared with BRAFposLTneg; (C) macrophages: GSEA: BRAFposLTpos compared with BRAFposLTneg. A full colour version of this figure is available at https://doi.org/10.1530/ERC-26-0088.

Pseudobulk comparative expression analysis found *DNASE1L3* (Log2FC 6.28, padj < 0.05) as the sole upregulated gene in LTpos macrophages (Fig. 4A and B). *DNASE1L3* is expressed in antigen-presenting cells and has been implicated in the regulation of immune tolerance and antitumour immunity, with reduced expression being associated with poorer outcomes across multiple cancer types (26).

In contrast, LTneg macrophages demonstrated upregulation of genes associated with immunosuppressive or alternatively activated macrophage states, including *USP53, COLEC12, CLEC5A, MRC1, WNT5A* and *TACSTD2* (Log2FC −2.65 to −4.31, padj < 0.05) (27, 28, 29, 30, 31, 32, 33). These transcriptional features are consistent with macrophage programmes linked to immune evasion and tumour progression. Further information on these genes involved is provided in Supplementary Table 3A and B.

Gene set enrichment analysis further supported functional divergence between groups, with the interferon gamma response (NES: 1.91, padj < 0.05) being enriched in LTpos macrophages, consistent with a more immune-effective macrophage phenotype in the presence of lymphocytic thyroiditis (Fig. 4C).

### Neutrophils in LTpos are associated with tumour inhibitory effect and in LTneg PTCs are associated with predominantly tumour-promoting effect

**Figure 5:**
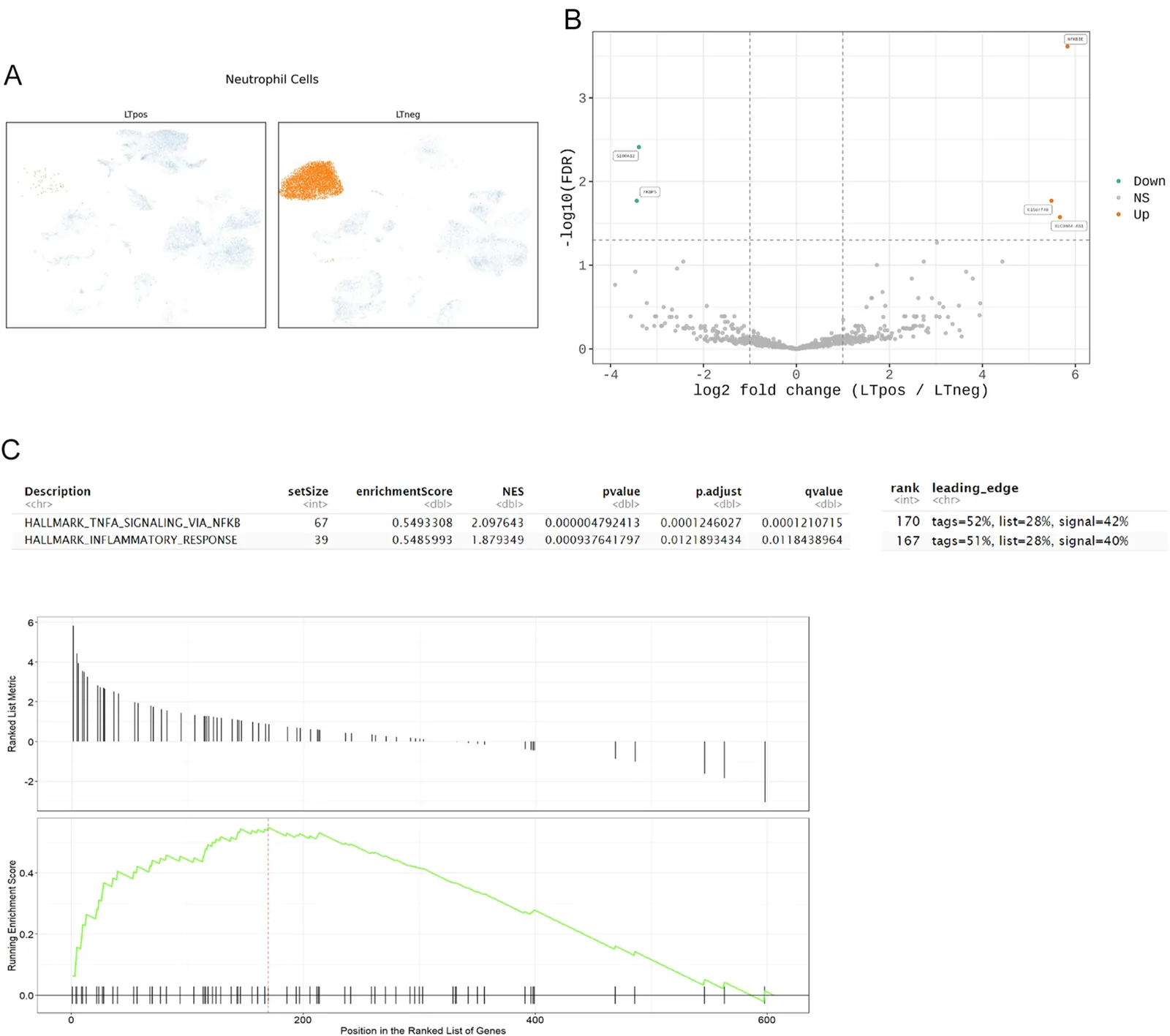
(A) Neutrophil cell clusters; (B) neutrophil volcano plots: BRAFposLTpos compared with BRAFposLTneg; (C) neutrophils: GSEA: BRAFposLTpos compared with BRAFposLTneg. A full colour version of this figure is available at https://doi.org/10.1530/ERC-26-0088.

LTpos neutrophils showed upregulation of *NFKBIE, C15ORF48* and *SLC30A4-S1* (Fig. 5A and B). Among these, *NFKKBIE*, a negative regulator of NF-κB signalling in myeloid cells, has been implicated in the suppression of tumour development (34). The functional roles of *C15ORF48 and SLC30A4-S1* remain undefined; however, their expression has previously been associated with immune infiltration (35, 36).

In contrast, LTneg neutrophils demonstrated increased expression of *S100A12* and *FKBP5,* which are linked to pro-tumourigenic and inflammatory neutrophil states and have been strongly correlated with thyroid cancer (37, 38). Further information on these genes involved is provided in Supplementary Table 4A and B.

Gene set enrichment analysis demonstrated significant activation of TNFα signalling via NF-κB (NES = 2.10, adjusted *P* < 0.05) and the Hallmark Inflammatory Response pathway (NES = 1.87, adjusted *P* < 0.05) in LTpos neutrophils (Fig. 5C). This inflammatory signature occurred within the context of lymphocytic thyroiditis and broader immune infiltration, consistent with a regulated, immune-effective inflammatory state rather than chronic tumour-promoting inflammation observed in LTneg tumours.

### Myeloid enrichment in LTneg PTCs confirmed by spatial transcriptomics and IHC

#### Spatial transcriptomics

Gene set module scoring was done with the Seurat and UCell R packages and demonstrated that neutrophils were enriched in the spatial feature plots in LTneg samples (Supplementary Fig. 1). However, it is noteworthy that spatial feature plots did not consistently exhibit neutrophils across all samples, likely due to the small dimensions of our tumour microarray.

#### Immunohistochemistry

**Figure 6:**
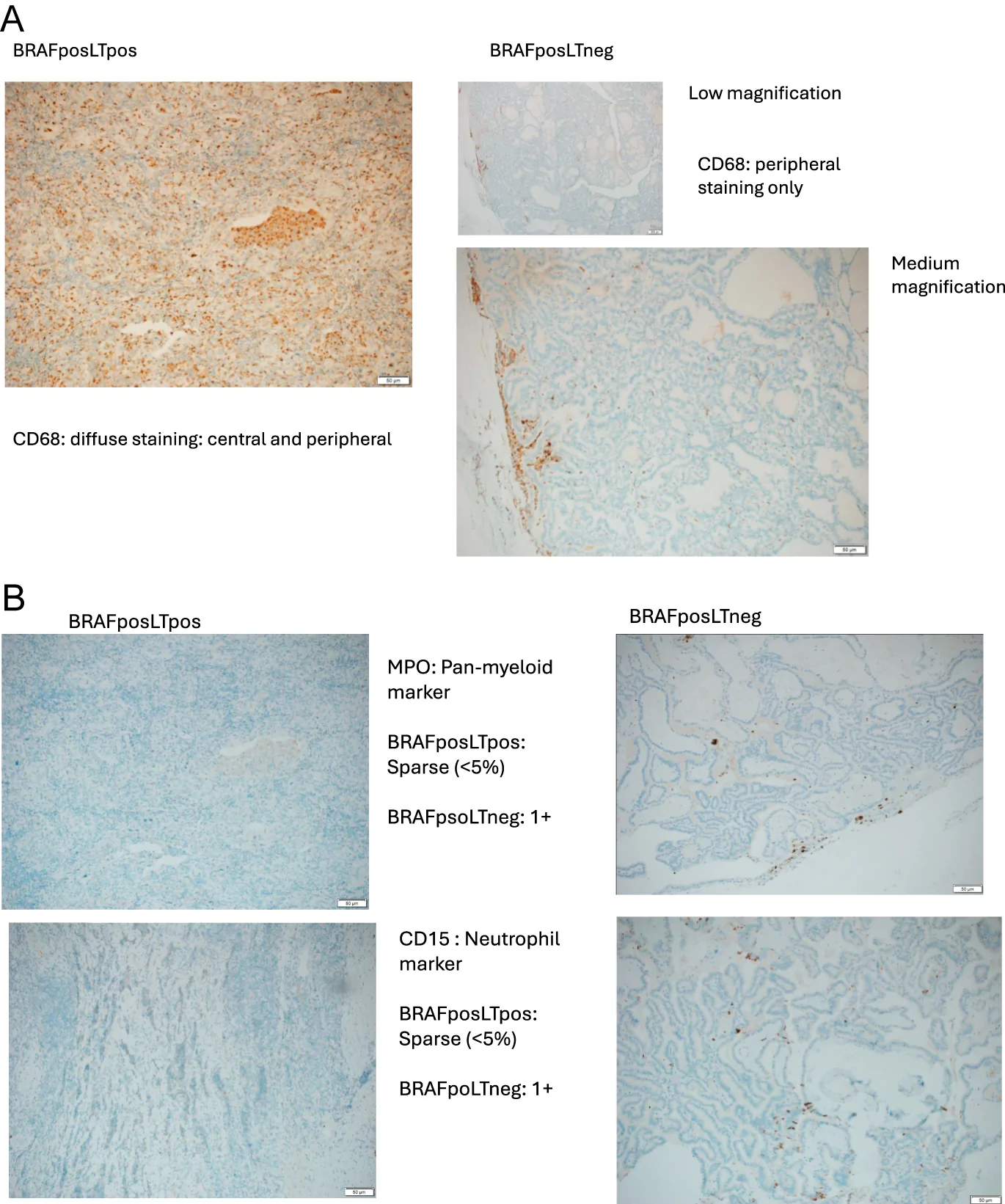
(A) Immunohistochemistry: CD68 macrophage marker; (B) immunohistochemistry: MPO and CD15. A full colour version of this figure is available at https://doi.org/10.1530/ERC-26-0088.

Cells expressing the macrophage marker CD68 were diffused in LTpos PTCs but predominantly peripherally located in LTneg PTCs (Fig. 6A and B). Similarly, the pan-myeloid marker MPO was also more prominent in LTneg PTCs with peripheral rather than central distribution, potentially at the leading edge of LTneg PTCs. Neutrophils were assessed by CD15 staining on IHC, although by semi-quantitation showed no significant difference between LTpos and LTneg PTCs (Supplementary Table 5).

## Discussion

In this study, TME composition was compared between PTC-BRAF with LT and PTC-BRAF without LT. This experimental design aimed to leverage a natural model of immune response against cancer to identify drivers and mechanisms. In particular, the role of myeloid cells in the TME of PTC-BRAF has not been well established. Here, we have thoroughly characterised the immune composition of PTC-BRAF samples, focussing on the myeloid cell lineage (macrophages and neutrophils) and highlighting differences between samples with and without coexisting LT. Our main findings were that neutrophils were significantly enriched in LTneg PTCs, whereas macrophages differed primarily in their distribution (rather than absolute numbers), being peripherally located in LTneg PTCs and diffusely located in LTpos PTCs.

Macrophages and T cells have previously been suggested to be the most common immune cell type in PTCs (39). Here, after carefully curating samples for the presence or absence of LT, we have shown that neutrophils are the predominant myeloid cell type in LTneg PTCs. In other cancers, neutrophils have been associated with progression, but there are limited data on their role in thyroid cancer (40, 41); some studies have found neutrophils to be associated with an increased risk of thyroid cancer recurrence and metastases (39, 42, 43). Means *et al.* showed that PTCs were generally myeloid-inflamed relative to adjacent non-malignant thyroid tissue using multiplex immunohistochemistry (44). The myeloid-rich tumours were associated with more aggressive pathological features. Our findings are consistent with these observations and extend them by resolving the myeloid compartment at single-cell resolution in a BRAF-mutant cohort stratified by LT status, identifying neutrophil enrichment and associated transcriptional programmes in LTneg tumours. We speculate that neutrophils may be actively recruited into PTCs (in the absence of LT), and in support of this, we found that LTneg thyrocytes showed significant expression of the neutrophil chemokine *ECRG4*. Neutrophils in the leading edge of tumours may recruit immunosuppressive M2-polarised TAMs and impair T cell infiltration (45). We demonstrated that in LTneg PTCs, myeloid cells occurred predominantly in the tumour periphery, whereas in LTpos PTCs, they were more diffuse throughout the tumour sample.

*S100A12* and *FKBP5* were upregulated in our LTneg neutrophils. Other studies have examined the role of *S100A12* in PTC: in one, *S100A12* was significantly upregulated in PTC and was associated with tumour size, tumour–node–metastasis (TNM) stage and lymph node metastasis (LNM) in PTCs and *S100A12* silencing markedly inhibited PTC cell proliferation, migration, invasion and cell cycle progression (46). In another study, although *S100A12* was identified in PTCs by multi-omics analysis, it was associated with protective effects by antagonising Treg activity (47). *FKBP5* expression was also associated with an increased incidence of intraglandular dissemination and a lower overall and progression-free survival (38). Conversely, LTpos neutrophils were associated with TNF-α via NF-κB and inflammatory response signalling as the predominant immune signalling pathways. Acute TNF-α exposure has been shown to stimulate immune activation and tumour cell death, but chronic high levels within the tumour microenvironment may drive immune exhaustion (48).

Increased antigen presentation is typically associated with improved immune surveillance. Our study found within LTpos thyrocyte samples a striking increase in the expression of *HLA-DQA1,* a gene involved in MHC presentation (https://www.proteinatlas.org/ENSG00000196735-HLA-DQA1; 13). MHC presentation may directly interact with CD4+ T-cells, which in turn have critical roles in supporting B cells and CD8+ T cell activation and generation of memory T cells (49). *HLA-DQA1* haplotypes are significantly increased in patients with LT (50). *HLA-DQA1*, MCH-II isotype, is driven by interferon gamma (IFNG)-induced transcriptional master regulator class II transactivator (CIITA) (14). Notably, all samples in our study were *BRAF-*mutated, allowing us to draw inferences on the effects of LT independent of *BRAF* activation. Thus, although oncogenic BRAF has been shown to suppress MHC-II expression in PTC through a TGF-β-dependent autocrine loop (51, 52), we regard the differences in MHC-II presentation to be more likely due to the presence of an IFNG-rich environment in LT (and therefore increased MHC-II expression) rather than downregulated MHC-II in LTneg samples. Specific alleles within MHC-II have been identified as critical in presenting neoantigens in various cancers contributing to effective antitumour immune response (53). MHC-II pathway genes show strong correlation with improved prognosis in breast cancer (54). Higher MHC-II presentation in PTC-BRAF with LT may therefore suggest an enhanced immune response.

Upregulation of IFNG response as the main signalling pathway in LTpos thyrocytes and macrophages was observed in our study. IFNG has direct effects on tumour cell immunogenicity by inducing tumour cell expression of MHC and thus plays an important role in promoting tumour cell recognition and elimination (55). However, in a chronic or low-level setting, IFNG signals can upregulate inhibitory pathways (e.g. PD-L1 and IDO1) and allow tumour cell to resist immunity (56). Thus, IFNG effect is dependent on the clinical context. In our LTpos samples, the TME is associated with strikingly enhanced IFNG signalling, which is associated with Th1 immune response (57).

Our findings are consistent with an association between coexisting LT and a less pro-tumourigenic tumour microenvironment in PTC-BRAF. LT was associated with enhanced antigen presentation and fewer neutrophils with immunosuppressive features. The observed differences in neutrophils between LTpos and LTneg samples may reflect an association between the interferon gamma-rich state of LT and reduced pro-tumourigenic innate immune activity within thyroid cancer. However, it remains unclear whether LT actively suppresses tumour progression or is preferentially maintained in less aggressive tumours. Accordingly, our findings demonstrate association rather than causation, and further longitudinal and mechanistic studies are required to clarify the temporal and biological relationship between LT and the observed tumour microenvironment.

There are a number of limitations to our study. We have deliberately focussed on myeloid cells in this study, since relatively little is known about the role of neutrophils in thyroid cancer (58). We intend to present data on adaptive immune cells separately in a subsequent paper. The observational nature of our work limits conclusions regarding causality: that is, we cannot be certain whether LT pre-existed the onset of thyroid cancer or if it was a cancer-evoked immune response. Furthermore, the relatively small cohort size represents an important limitation of this study. The modest sample size reflects the limited availability of freshly resected BRAF-mutant PTC specimens with coexisting LT suitable for single-cell RNA sequencing. Importantly, the principal findings were consistently observed across patients within each group and were independently corroborated by both spatial transcriptomics and immunohistochemistry, supporting the robustness of our observations despite the modest cohort size. Accordingly, our findings should be considered hypothesis-generating and require validation in larger, independent cohorts. Batch effects and technical variability due to differences in cell capture and sequencing cannot be excluded. As a study of cells that are naturally dynamic, but captured at one moment in time, this may not have fully represented biological heterogeneity defined by marker genes. Validation of specific cell types and their function requires further experimental evaluation.

## Conclusion

Improved outcomes seen in PTC-BRAF with LT led us to leverage this natural model of effective adaptive immune response to evaluate how LT changes the TME in PTC-BRAF using single-cell RNA sequencing. When PTC-BRAF was accompanied by LT, we found upregulation of MHC-II antigen presentation in thyrocytes. In PTC-BRAF without LT, there was predominance of neutrophils driven by pro-tumourigenic genes. Key properties of these innate immune cells were associated with the presence or absence of LT, suggesting new mechanisms to target immune treatments in PTC refractory to other therapies.

## Supplementary materials





















## Declaration of interest

The authors declare that there is no conflict of interest that could be perceived as prejudicing the impartiality of the work reported.

## Funding

This work was supported by the Ramsay Research and Teaching Fund 2023.

## Ethics

This study was approved by the Northern Sydney Health District Research Ethics Committee (2023/ETH00856). All patients provided written informed consent after full explanation of the purpose and nature of all procedures used.
