# Supplementary material for: Myeloid landscape of BRAF-mutant papillary thyroid cancer and thyroiditis
Source: Endocr Relat Cancer. 2026 Aug 31;33(8):e260088. doi: 10.1530/ERC-26-0088 (PMC13539360; doi:10.1530/ERC-26-0088)

Supplementary Figure 1: Spatial Transcriptomics

Spatial Feature Plots representing Ucell Module score for cell types

Neutrophil: BRAFposLTpos

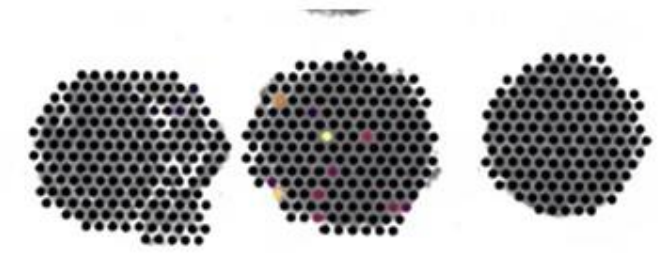

Neutrophil: BRAFposLTneg

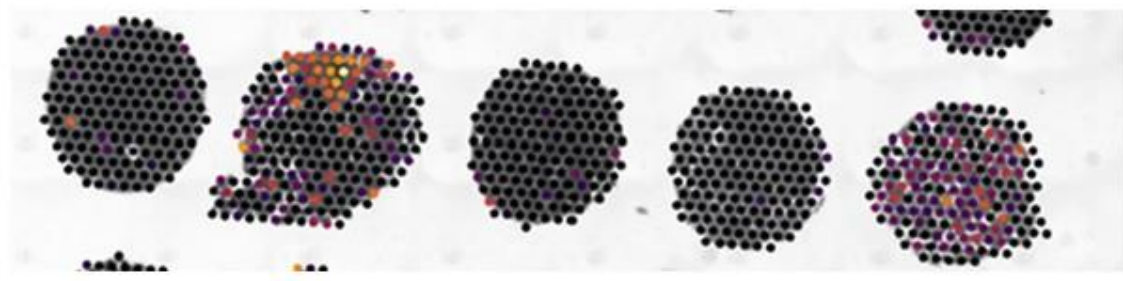

Macrophage: BRAFposLTpos

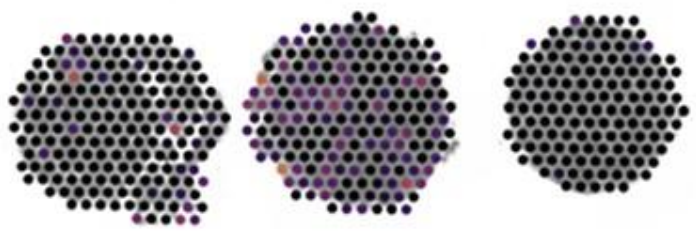

Macrophage:BRAFposLTneg

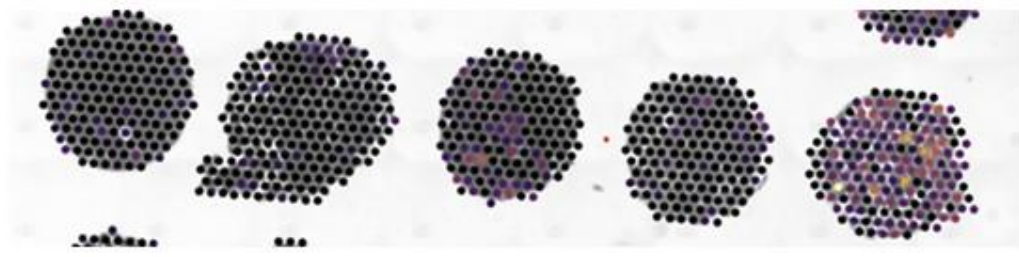

Supplement: Supplementary file 1 [file ERC-26-0088_supplementary_figure_1.pdf]
