# Supplementary material for: Myeloid landscape of BRAF-mutant papillary thyroid cancer and thyroiditis
Source: Endocr Relat Cancer. 2026 Aug 31;33(8):e260088. doi: 10.1530/ERC-26-0088 (PMC13539360; doi:10.1530/ERC-26-0088)
Supplement: Supplementary file 2 [file ERC-26-0088_supplementary_materials.pdf]

Supplementary References:

Table 2a: Thyroid epithelial cells : BRAFposLTpos compared to BRAFposLTneg

-upregulated genes

(1-6)

Table 2b: Thyroid epithelial cells : BRAFposLTpos compared to BRAFposLTneg

-downregulated genes

(7-14)

Table 3a: Macrophage: BRAFposLTpos compared to BRAFposLTneg

-upregulated genes

(15)

Table 3b: Macrophage: BRAFposLTpos compared to BRAFposLTneg

-downregulated genes

(16-21)

Table4a: Neutrophils: BRAFposLT pos compared to BRAFposLTneg

-upregulated genes

(22-24)

Table4b: Neutrophils: BRAFposLT pos compared to BRAFposLTneg

-downregulated genes

(25, 26)

References:

1. LTB lymphotoxin beta [Homo sapiens (human)] Gene ID: 4050 [Internet]. Available from: https://www.ncbi.nlm.nih.gov/gene/4050#:~:text=LTB%20is%20an%20inducer%20of,is%20independent%20of%20lympyhotoxin%2Dalpha.
2. Su H, Na N, Zhang X, Zhao Y. The biological function and significance of CD74 in immune diseases. Inflamm Res. 2017;66(3):209-16.
3. Steimle V, Siegrist CA, Mottet A, Lisowska-Grospierre B, Mach B. Regulation of MHC class II expression by interferon-gamma mediated by the transactivator gene CIITA. Science. 1994;265(5168):106-9.
4. The Human Protein Atlas: HLA-DQA1.
5. Vilagos B, Hoffmann M, Souabni A, Sun Q, Werner B, Medvedovic J, et al. Essential role of EBF1 in the generation and function of distinct mature B cell types. J Exp Med. 2012;209(4):775-92.
6. Human Protein Atlas: LAT2 [Internet]. Available from: https://www.proteinatlas.org/ENSG00000086730-LAT2.
7. Tan Y, Chen Q, Pan S, An W, Xu H, Xing Y, et al. LMOD1, an oncogene associated with Lauren classification, regulates the metastasis of gastric cancer cells through the FAK-AKT/mTOR pathway. BMC Cancer. 2022;22(1):474.

8. Jian Y, Kong L, Xu H, Shi Y, Huang X, Zhong W, et al. Protein phosphatase 1 regulatory inhibitor subunit 14C promotes triple-negative breast cancer progression via sustaining inactive glycogen synthase kinase 3 beta. Clin Transl Med. 2022;12(1):e725.
9. Hareendran S, Yang X, Sharma VK, Loh YP. Carboxypeptidase E and its splice variants: Key regulators of growth and metastasis in multiple cancer types. Cancer Lett. 2022;548:215882.
10. Przygodzka P, Szulc-Kielbik I, Kielbik M, Pacholczyk M, Klink M. Neuromedin U in the tumor microenvironment - Possible actions in tumor progression. Biochim Biophys Acta Rev Cancer. 2025;1880(2):189269.
11. Elsheikh SE, Green AR, Rakha EA, Samaka RM, Ammar AA, Powe D, et al. Caveolin 1 and Caveolin 2 are associated with breast cancer basal-like and triple-negative immunophenotype. Br J Cancer. 2008;99(2):327-34.
12. Alnwisser B, Alshehri S, Qattan A, Zou M, Aboussekhra A, Alzahrani AS, et al. Identification of novel prognostic biomarkers for thyroid cancer by integrated transcriptome analysis of metastasis-associated genes. Front Oncol. 2025;15:1536270.
13. Chen J, Liu C, Yin L, Zhang W. The tumor-promoting function of ECRG4 in papillary thyroid carcinoma and its related mechanism. Tumour Biol. 2015;36(2):1081-9.
14. Dorschner RA, Lee J, Cohen O, Costantini T, Baird A, Eliceiri BP. ECRG4 regulates neutrophil recruitment and CD44 expression during the inflammatory response to injury. Sci Adv. 2020;6(11):eaay0518.
15. Li W, Nakano H, Fan W, Li Y, Sil P, Nakano K, et al. DNASE1L3 enhances antitumor immunity and suppresses tumor progression in colon cancer. JCI Insight. 2023;8(17).
16. Liu Y, Tang W, Yao F. USP53 Exerts Tumor-Promoting Effects in Triple-Negative Breast Cancer by Deubiquitinating CRKL. Cancers (Basel). 2023;15(20).
17. Sun X, Zhang Q, Shu P, Lin X, Gao X, Shen K. COLEC12 Promotes Tumor Progression and Is Correlated With Poor Prognosis in Gastric Cancer. Technol Cancer Res Treat. 2023;22:15330338231218163.
18. Chen R, Wu W, Chen SY, Liu ZZ, Wen ZP, Yu J, et al. A Pan-Cancer Analysis Reveals CLEC5A as a Biomarker for Cancer Immunity and Prognosis. Front Immunol. 2022;13:831542.
19. Wu Z, Huang C. Unveiling the Impact of MRC1 on Immune Infiltration and Patient's Prognosis: A Pan-Cancer Analysis Based on Single-Cell and Bulk Sequencing. Int J Gen Med. 2024;17:2575-92.
20. Bueno MLP, Saad STO, Roversi FM. WNT5A in tumor development and progression: A comprehensive review. Biomed Pharmacother. 2022;155:113599.
21. Ning N, Lu J, Li Q, Li M, Cai Y, Wang H, et al. Single-sEV profiling identifies the TACSTD2 + sEV subpopulation as a factor of tumor susceptibility in the elderly. J Nanobiotechnology. 2024;22(1):222.
22. Xia Y, Shen S, Verma IM. NF-kappaB, an active player in human cancers. Cancer Immunol Res. 2014;2(9):823-30.
23. Ren J, Zhu H, Liu M, Zhang R, Mao W, Xiao S. C15ORF48 serves as a potential biomarker and therapeutic target in pan-cancer with implications for lung cancer. Sci Rep. 2025;15(1):32821.
24. Guo Y, He Y. Comprehensive analysis of the expression of SLC30A family genes and prognosis in human gastric cancer. Sci Rep. 2020;10(1):18352.
25. Wang X, Sun Z, Tian W, Piao C, Xie X, Zang J, et al. S100A12 is a promising biomarker in papillary thyroid cancer. Sci Rep. 2020;10(1):1724.
26. Gao Z, Yu F, Jia H, Ye Z, Yao S. FK506-binding protein 5 promotes the progression of papillary thyroid carcinoma. J Int Med Res. 2021;49(4):3000605211008325.
