# Supplementary material for: Myeloid landscape of BRAF-mutant papillary thyroid cancer and thyroiditis
Source: Endocr Relat Cancer. 2026 Aug 31;33(8):e260088. doi: 10.1530/ERC-26-0088 (PMC13539360; doi:10.1530/ERC-26-0088)
Supplement: Supplementary file 3 [file ERC-26-0088_supplementary_table_1.pdf]

Table1: Samples with histopathological features, cell numbers, gene expression processing chemistry

Sample_ID	Sex	BRAF pos	LT Pos Neg	Age	TNM	Size(mm)	LN disease	ETE	LVI	Cell_Number	Chemistry
DM-2294394	M	pos	neg	43	T1N1aM0	8	pos	neg	pos	2,258	Single Cell 3' v3
KL-2282851	F	pos	neg	38	T1bN0M0	15	neg	neg	pos	6,590	Single Cell 3' v3
MA-2295339	M	pos	neg	29	T3aN1b	47	pos	pos	pos	5,208	Single Cell 3' v3
MG-2295633	F	pos	neg	38	T1N0M0	9	neg	neg	neg	13,536	Single Cell 3' v4
MT-2271260	F	pos	neg	61	T1N0M0	12	neg	pos	neg	15,758	Single Cell 3' v4
VF-1814538	M	pos	neg	39	T2N1bM0	30	pos	pos	pos	5,062	Single Cell 3' v3
AW-2347516	M	pos	neg	33	T2N1bM0	36	pos	pos	pos	9,922	Flex Gene Expression
JW-0445908	F	pos	pos	73	T1mN0	9	neg	neg	neg	18,878	Single Cell 3' v3
SK-1450222	F	pos	pos	19	T1N1aM0	18	pos	neg	neg	5,048	Single Cell 3' v3
SD-00462242	F	pos	pos	34	T1N0M0	20	neg	neg	pos	20,334	Flex Gene Expression
gse281736	F	pos	pos	29	T2N1bM0	35	pos	neg	not stated	not stated	Single Cell 3' v3

Total cells: 108,885
BRAFposLTpos= 50,677
BRAFposLTneg= 58,208
