# Supplementary material for: Myeloid landscape of BRAF-mutant papillary thyroid cancer and thyroiditis
Source: Endocr Relat Cancer. 2026 Aug 31;33(8):e260088. doi: 10.1530/ERC-26-0088 (PMC13539360; doi:10.1530/ERC-26-0088)
Supplement: Supplementary file 4 [file ERC-26-0088_supplementary_table_2a.pdf]

Supplementary Table 2a: Thyroid cells: BRAFposLTpos compared to BRAFposLTneg

Upregulated Genes	Function	Reference
LTB	LTB is an inducer of the inflammatory response system and involved in normal development of lymphoid tissue.	https://www.ncbi.nlm.nih.gov/gene/4050#:~:text=LTB%20is%20an%20inducer%20of,is%20independent%20of%20lympyhotoxin%2Dalpha.
CD74	CD74 (MHC class II invariant chain, Ii) is a non-polymorphic type II transmembrane glycoprotein. CD74 is a cell membrane high-affinity receptor for macrophage migration inhibitory factor (MIF), also regulates T-cell and B-cell developments, dendritic cell (DC) motility, macrophage inflammation, and thymic selection.	(Su, Na et al. 2017) doi: 10.1007/s00011-016-0995-1.
CIITA	A functional CIITA gene is necessary for class II induction, and transfection of CIITA is sufficient to activate expression of MHC class II genes in class II-negative cells in the absence of IFN-gamma. CIITA is therefore a general regulator of both inducible and constitutive MHC class II expression.	(Steimle, Siegrist et al. 1994) doi: 10.1126/science.8016643
HLADQA1	Major histocompatibility complex, class II, HLADQ alpha 1, immune proteins which binds peptides derived from antigens and presents them on the cell surface for recognition by the CD4 T-cells.	doi.org/10.1038/gene.2008.26<br><br>https://www.proteinatlas.org/ENSG00000196735-HLA-DQA1
EBF1	The transcription factor EBF1 is essential for lineage specification in early B cell development. These gain- and loss-of-function analyses uncovered novel important functions of EBF1 in controlling B cell immunity	(Vilagos, Hoffmann et al. 2012) doi: 10.1084/jem.20112422
LAT2	Involved in FCER1 (high affinity immunoglobulin epsilon receptor)-mediated signaling in mast cells. May also be involved in BCR (B-cell antigen receptor)-mediated signaling in B-cells and FCGR1 (high affinity immunoglobulin gamma Fc receptor I)-mediated signaling in myeloid cells.	Human Protein Atlas: LAT2 [Internet]. Available from: https://www.proteinatlas.org/ENSG00000086730-LAT2.
