# Supplementary material for: Myeloid landscape of BRAF-mutant papillary thyroid cancer and thyroiditis
Source: Endocr Relat Cancer. 2026 Aug 31;33(8):e260088. doi: 10.1530/ERC-26-0088 (PMC13539360; doi:10.1530/ERC-26-0088)
Supplement: Supplementary file 5 [file ERC-26-0088_supplementary_table_2b.pdf]

Supplementary Table 2b: Thyroid epithelial cells: BRAFposLTpos compared to BRAFposLTneg

**Down regulated genes**	**Function**	**Reference**
LMOD1	LMOD1 is an oncogene associated with diffuse gastric cancer and can affect the occurrence and development of EMT by regulating the FAK-Akt/mTOR pathway. LMOD1 can therefore promote peritoneal metastasis of gastric cancer cells and can be used as a novel therapeutic target for gastric cancer.	(Tan, Chen et al. 2022)<br>doi: 10.1186/s12885-022-09541-0
PPP1R14C	Protein phosphatase 1 regulatory inhibitor subunit 14C (PPP1R14C) is upregulated in TNBC and relevant to poor prognosis in patients. Overexpression of PPP1R14C facilitates cell proliferation and the aggressive phenotype of TNBC cells, whereas the depletion of PPP1R14C elicits opposite effects.	(Jian, Kong et al. 2022)<br>DOI: 10.1002/ctm2.725
CPE	CPE enriched in endocrine tumors, found to be expressed and secreted from many epithelial-derived tumors and cancer cell lines. Numerous studies have shown that besides wild-type CPE, a N-terminal truncated splice variant form of CPE (CPE-ΔN) has been cloned and found to be highly expressed in malignant tumors and cell lines derived from prostate, breast, liver and lung cancers and gliomas. CPE and the splice variant promotes tumor growth and metastasis in different cancer types.	(Hareendran, Yang et al. 2022)<br>DOI: 10.1016/j.canlet.2022.215882
NMU	Expression of NMU resulted in significantly enhanced both cell migration and invasion of renal cancer cells with no effect on proliferation. NMU level in the tumor tissue was significantly correlated with the expression of genes coding CD4, CD25, FOXP3, NFAT, IL-4, and IL-13, suggesting that peptide may activate Tregs, as well as induce TAM-M2 polarization. NMU is a mediator of interactions between cancer and immune cells and in that way may influence the progression of cancer.	(Przygodzka, Szulc-Kielbik et al. 2025)<br>DOI: 10.1016/j.bbcan.2025.189269

CAV1, CAV2	Caveolin-1 and CAV2 were expressed in invasive breast cancer cases, respectively, and were significantly associated with high histological grade, lack of hormone receptor positivity, and a basal-like phenotype. While CAV2 had a prognostic impact on breast cancer-specific survival, this was not independent from other traditional markers.	(Elsheikh, Green et al. 2008)<br>DOI: 10.1038/sj.bjc.6604463
TENM3	Seven genes were identified, and their overexpression was associated with poor OS (ARG1, TF, CD52, ITGBL1, PTGIR, TENM3, and AS3MT) in PTC. The seven-gene expression signature is associated with poor prognosis and chromosomal instability.	(Alnwisser, Alshehri et al. 2025)<br>doi: 10.3389/fonc.2025.1536270
ECRG4	The expression of ECRG4 is frequently upregulated in a papillary thyroid carcinoma through the demethylation mechanism of CpG islands in the gene promoter region, and the ECRG4 has a tumor-promoting function through inducing the cell cycle transition from the G1 phase to the G2 in papillary thyroid carcinoma cells.<br>ECRG4 regulates early neutrophil recruitment.	(Chen, Liu et al. 2015)<br>doi: 10.1007/s13277-014-2731-1<br>(Dorschner, Lee et al. 2020)<br>doi: 10.1126/sciadv.aay0518
