# Supplementary material for: Myeloid landscape of BRAF-mutant papillary thyroid cancer and thyroiditis
Source: Endocr Relat Cancer. 2026 Aug 31;33(8):e260088. doi: 10.1530/ERC-26-0088 (PMC13539360; doi:10.1530/ERC-26-0088)
Supplement: Supplementary file 6 [file ERC-26-0088_supplementary_table_3a.pdf]

Supplementary Table 3a: Macrophage: BRAFposLTpos compared to BRAFposLTneg

**Upregulated Genes**	**Function**	**References**
DNASE1L3	DNASE1L3 as a potentially new regulator of antitumor immunity and a tumor suppressor in colon cancer. In humans, *DNASE1L3* is downregulated in tumor-infiltrating DCs, and this downregulation is associated with poor patient prognosis and reduced tumor immune cell infiltration in many cancer types.	(Li, Nakano et al. 2023)<br>DOI:10.1172/jci.insight.168161
