# Supplementary material for: Myeloid landscape of BRAF-mutant papillary thyroid cancer and thyroiditis
Source: Endocr Relat Cancer. 2026 Aug 31;33(8):e260088. doi: 10.1530/ERC-26-0088 (PMC13539360; doi:10.1530/ERC-26-0088)
Supplement: Supplementary file 7 [file ERC-26-0088_supplementary_table_3b.pdf]

Supplementary 3b: Macrophage: BRAFposLTpos compared to BRAFposLTneg

**Downregulated Genes**	**Function**	**Reference**
USP53	USP53, one of ubiquitin-specific peptidase (USP) family members, widely expressed in human tissues and participates in cell apoptosis, nerve transmission, and bone remodeling.USP53 plays a tumor-suppressive role in lung cancer, renal clear cell carcinoma, colorectal cancer, liver cancer, and esophageal cancer	(Liu, Tang et al. 2023)<br>DOI: 10.3390/cancers15205033
COLEC12	COLEC12 functions as a pattern-recognition molecule initiating complement activation through the alternative pathway. COLEC12 expression was moderately to strongly correlated with the expression of M2 macrophage, involved in several tumor processes, and metastasis in breast and gastric cancer.	(Sun, Zhang et al. 2023)<br>doi: 10.1177/15330338231218163
CLEC5A	CLEC5A, member of the C-type lectin superfamily, can activate macrophages and lead to a series of immune-inflammation reactions.*CLEC5A* is differentially expressed in a few cancer types, of which overexpression accompanies low overall survival of patients.	(Chen, Wu et al. 2022)<br>doi: 10.3389/fimmu.2022.831542
MRC1	MRC1 plays an important role in antigen presentation and the induction of immune response; a significant marker protein expressed in M2; role as an essential signature within this pro-tumorigenic environment. MRC1 is an unfavorable factor regarding overall survival in GBM, KICH, LAML, LUSC, THCA, and THYM patients.	(Wu and Huang 2024)<br>doi: 10.2147/IJGM.S461144

WNT5A	WNT5A, an evolutionary WNT member, is involved in several cellular and physiopathological processes. The WNT5A protein exerts paradoxical effects both an oncogene or tumor suppressor by regulating several non-canonical signaling pathways, and consequently interfering in cell growth, cytoskeletal remodeling, migration and invasiveness.	(Bueno, Saad et al. 2022)<br>doi.org/10.1016/j.biopha.2022.113599
TACSTD2	9 of the 13 tumor groups exhibited elevated TACSTD2, particularly for cervical cancer, colon cancer, esophageal carcinoma, liver cancer and thyroid carcinoma. Those with high TACSTD2 levels promoted tumor cell (SW480, HuCCT1 and HeLa) proliferation and migration.	(Ning, Lu et al. 2024)<br>doi: 10.1186/s12951-024-02456-x
