# Supplementary material for: Myeloid landscape of BRAF-mutant papillary thyroid cancer and thyroiditis
Source: Endocr Relat Cancer. 2026 Aug 31;33(8):e260088. doi: 10.1530/ERC-26-0088 (PMC13539360; doi:10.1530/ERC-26-0088)
Supplement: Supplementary file 8 [file ERC-26-0088_supplementary_table_4a.pdf]

Supplementary Table 4a: Neutrophils: BRAFposLT pos compared to BRAFposLTneg

**Upregulated Genes**	**Function**	**Reference**
NFKBIE	NF-κB inhibition in different organs has varying effects on<br>Tumorigenesis; the inhibition of NF-κB in inflammatory or myeloid cells consistently suppresses tumor development. NF-κB activation in myeloid cells typically enhances inflammation in the tumor microenvironment by increasing the secretion of pro-inflammatory cytokines such as TNF-α and IL-6, which eventually leads to rapid proliferation of tumor cells	10.1158/2326-6066.CIR-14-0112.
C15ORF48	C15ORF48 expression is generally elevated in tumor versus normal tissues; higher C15ORF48 expression is associated with poorer survival in lung adenocarcinoma. Genetic alterations in *C15orf48* are identified across various cancers. C15ORF48 could play a significant role in pan-cancer, particularly in lung cancer, and may serve as a potential biomarker for prognosis and targeted therapies.	(Ren, Zhu et al. 2025)<br>doi.org/10.1038/s41598-025-17966-3
SLC30A4-S1	*SLC30A1-7*, and *9* were significantly correlated with advanced tumor stage and nodal metastasis status; upregulated *SLC30A2-4*, *8*, and *10* expression was markedly associated with poor OS, FP and PPS. And SLC30A family genes were closely associated with the infiltration of immune cells.	(Guo and He 2020)<br>doi.org/10.1038/s41598-020-75012-w
