# Supplementary material for: Myeloid landscape of BRAF-mutant papillary thyroid cancer and thyroiditis
Source: Endocr Relat Cancer. 2026 Aug 31;33(8):e260088. doi: 10.1530/ERC-26-0088 (PMC13539360; doi:10.1530/ERC-26-0088)
Supplement: Supplementary file 9 [file ERC-26-0088_supplementary_table_4b.pdf]

Supplementary Table 4b: Neutrophils: BRAFposLT pos compared to BRAFposLTneg

Downregulated Genes	Function	Reference
S100A12	S100 proteins perform a large range of functions, including regulating cell proliferation and invasion, interacting with the cytoskeleton interplays, and mediating transcription and inflammation. S100A12 was significantly upregulated in PTC specimens; significantly increased and is associated with clinicopathological characteristics in human PTC samples, tumor size, tumor-node-metastasis (TNM) stage and lymph node metastasis (LNM).Moreover, silencing S100A12 markedly inhibited PTC cell proliferation, migration, invasion and cell cycle progression	(Wang, Sun et al. 2020)<br>doi.org/10.1038/s41598-020-58534-1
FKBP5	FKBP5 has involvement in the progression of multiple cancers. High expression levels of FKBP5 in human PTC tissues compared with normal tissues. Furthermore, high FKBP5 expression was associated with an increased incidence of intraglandular dissemination, and lower overall and progression-free survival.	(Gao, Yu et al. 2021)<br>doi:10.1177/03000605211008325
