# Supplementary material for: Myeloid landscape of BRAF-mutant papillary thyroid cancer and thyroiditis
Source: Endocr Relat Cancer. 2026 Aug 31;33(8):e260088. doi: 10.1530/ERC-26-0088 (PMC13539360; doi:10.1530/ERC-26-0088)
Supplement: Supplementary file 10 [file ERC-26-0088_supplementary_table_5.pdf]

Table 5: Immunohistochemistry: samples and myeloid markers

Sample_ID	Age	Gender	BRAFpos	LTpos	CD68-Macropha	central_peripheral	CD15-Neutro	MPO-pan_myeloid	Histo -Size	Multifocal	LN	LVI	ETE	Margin Positive
132710	70	F	Yes	Yes	2+	mixed	sparse	sparse	8mm	No	Yes	No	No	No
48808	57	F	Yes	Yes	1+	central predom	2+	sparse	11mm	Yes	No	No	No	Yes
11053064	44	F	Yes	Yes	2+	central predom	0	neg	40mm	Yes	Yes	Yes	No	No
434821	44	F	Yes	Yes	1+	central predom	2+	neg	17mm	No	Yes	No	No	Yes
483871	59	F	Yes	Yes	1+	central predom	2+	neg	12mm	No	No	No	No	No
20582508	28	F	Yes	Yes	2+	central predom	2+	neg	16mm	No	Yes	No	No	No
712619	64	F	Yes	No	0	NAD	0	neg	15mm	Yes	Yes	No	No	No
20519142	33	M	Yes	No	1+	peripheral only	0	sparse	25mm	No	Yes	Yes	No	No
11052742	57	M	Yes	No	2+	peripheral only	2+	neg	36mm	No	No	No	no	no
20534353	29	M	Yes	No	2+	peripheral only	0	1+	23mm	Yes	Yes	No	No	No
30566632	56	M	Yes	No	2+	peripheral only	2+	neg	28mm	No	Yes	No	No	No
20173147	39	F	Yes	No	2+	peripheral only	1+	1+	27mm	No	Yes	No	No	No
